# Local staging of de novo prostate cancer using mpMRI, PSMA-PET and PSMA-PET/mpMRI – a comparative study

**DOI:** 10.1186/s13550-025-01334-3

**Published:** 2025-11-17

**Authors:** Josefine Grefve, Sara N. Strandberg, Joakim Jonsson, Angsana Keeratijarut Lindberg, Erik Nilsson, Anders Bergh, Karin Söderkvist, Camilla Thellenberg Karlsson, Lennart Nedar, Vibeke Berg Løgager, Erik Thimansson, Elin Trägårdh, Johan Bengtsson, Erland Hvittfeldt, Jan Axelsson, Tufve Nyholm, Katrine Riklund, Kristina Sandgren

**Affiliations:** 1 Department of Diagnostics and Intervention, Radiation Physics, Umeå University, Umeå, Sweden; 2 Department of Diagnostics and Intervention, Diagnostic Radiology, Umeå University, Umeå, Sweden; 3 Department of Medical Biosciences, Pathology, Umeå University, Umeå, Sweden; 4 Department of Diagnostics and Intervention, Oncology, Umeå University, Umeå, Sweden; 5 Department of Nuclear Medicine and Hospital Physics, Karolinska University Hospital, Stockholm, Sweden; 6 Department of Radiology, Copenhagen University Hospital in Herlev, Herlev, Denmark; 7 Department of Translational Medicine, Diagnostic Radiology, Lund University, Malmö, Sweden; 8 Department of Radiology, Helsingborg Hospital, Helsingborg, Sweden; 9 Department of Translational Medicine, Wallenberg Centre for Molecular Medicine, Lund University, Malmö, Sweden; 10 Department of Medical Imaging and Physiology, Skåne University Hospital, Lund, Sweden; 11 Department of Clinical Sciences, Diagnostic Radiology, Lund University, Lund, Sweden

## Abstract

**Background:**

Accurate diagnosis and staging are essential for optimal treatment planning of prostate cancer. By combining functional and anatomical imaging, PSMA-PET/mpMRI offers a potential to improve lesion detection and enhance staging accuracy. This study aimed to evaluate the diagnostic performance of lesion detection and local staging of prostate cancer using combined PSMA-PET/mpMRI compared to standalone mpMRI or PSMA-PET.

**Results:**

Fifty-five patients with intermediate- to high-risk prostate cancer scheduled for robot-assisted laparoscopic radical prostatectomy were included. All patients underwent [^68^Ga]PSMA-PET/mpMRI prior to surgery. Whole-mount histopathology and surgical report served as reference standard. Two radiologists independently evaluated mpMRI, while two nuclear medicine physicians assessed PSMA-PET. For the PSMA-PET/mpMRI analysis, a consensus evaluation was performed by a new set of readers in two teams, each comprising one radiologist and one nuclear medicine physician. Lesion localization was reported based on the PI-RADS v2.1 sector map and compared to histopathology. Among 130 histopathologically confirmed lesions, mean detection rates were 38% (49.5/130) for PSMA-PET/mpMRI, 32% (41/130) for mpMRI and 32% (41/130) for PSMA-PET. For clinically significant prostate cancer (csPC) (≥0.5 ml, ≥ISUP 2; 42 lesions), mean detection rates were 85% (35.5/42) for PSMA-PET/mpMRI, 75% (31.5/42) for mpMRI and 70% (29.5/42) for PSMA-PET. The mean false discovery rates were 8% (PSMA-PET/mpMRI), 15% (mpMRI) and 12% (PSMA-PET). The likelihood of extraprostatic extension (EPE) and seminal vesicle invasion (SVI) were scored using a 5-point Likert scale, where scores of 1–3 were classified as negative and scores of 4–5 were considered positive. Sensitivity for EPE was 32% for PSMA-PET/mpMRI, 37% for mpMRI and 7% for PSMA-PET, with a specificity of 100%, 96% and 98%, respectively. For SVI, sensitivity was 50% for PSMA-PET/mpMRI and 38% for mpMRI and PSMA-PET, with a specificity of 100%, 95% and 97% respectively.

**Conclusions:**

PSMA-PET/mpMRI provided higher and a more consistent performance in localized prostate cancer detection and staging without increasing false-positive findings.

## Introduction

Prostate cancer is one of the most prevalent malignancies in men, and accurate diagnosis and staging are critical for optimal treatment planning [1]. The TNM staging system was first introduced in the 1940s as a diagnostic tool for prostate cancer, based on tumour size, nodal involvement, and metastatic spread [2]. Over time, it has evolved into the classification system used today [3]. However, discrepancies between preoperative TNM staging and post-operative histological assessment are not uncommon [4]. One of the challenges in achieving accurate T-staging is the difficulty in detecting extraprostatic extension (EPE) and seminal vesicle invasion (SVI) particularly given that prostate cancer is often multifocal [5].

Prostate cancers classified as T3 are associated with an elevated risk of biochemical recurrence, positive surgical margins and metastasis [6, 7]. Consequently, the presence of EPE or SVI plays a critical role in treatment decision-making, including the selection of the most appropriate therapeutic approach, whether surgery, radiation, or surveillance, and in the modification of treatment strategies such as finding those that are eligible for nerve-sparing surgery [7, 8].

Over recent years, multiparametric MRI (mpMRI) has become a widely used imaging modality for detecting and assessing the extent of the disease due to its high soft-tissue contrast [9]. The Prostate Imaging Reporting and Data System (PI-RADS) guideline [10] is a widely used framework aiding in image acquisition, interpretation, staging and detection of clinically significant prostate cancer (csPC) using mpMRI. It is primarily designed for detection rather than defining the full tumour extent. mpMRI alone may not fully capture the entire tumour burden, a limitation that has been highlighted in multiple studies [11–13].

Prostate-specific membrane antigen (PSMA) ligands can be labelled with either [^68^Ga] or [^18^F] for diagnostic use in positron emission tomography (PET), usually in combination with computed tomography (PET/CT). PSMA-PET/CT has emerged as an established imaging modality in prostate cancer, where PET can give additional information to mpMRI regarding the PSMA expression of tumours [14]. The PRIMARY trial demonstrated that combining PSMA-PET/CT with mpMRI improves both sensitivity and negative predictive value in the diagnosis of prostate cancer [15, 16]. By integrating functional and anatomical imaging, PSMA-PET combined with mpMRI (PSMA-PET/mpMRI) has the potential to further enhance the detection of prostate cancer lesions [17, 18]. The ability to detect and accurately localize intraprostatic lesions is essential not only for guiding targeted biopsies but also for radiotherapy planning, where dose escalation to the dominant intraprostatic lesion has become increasingly used.

Regarding local staging accuracy, a recent study reported an advantage of using MRI over PSMA-PET/CT for detecting EPE [19]. In contrast, other studies have showed higher performance for PSMA-PET compared to mpMRI in predicting EPE [20, 21]. Another study showed that PSMA-PET/MRI provided a reliable TNM staging of localized prostate cancer [22]. They also present a clinical benefit of using PSMA-PET/MRI in localized prostate cancer, with one third of the patients included in the study having a change in the therapeutic management based on the PSMA-PET/MRI.

Based on current literature, PSMA-PET/MRI appears to be a promising modality for both lesion detection and local staging in prostate cancer. However, further evidence is needed to establish its diagnostic accuracy and reproducibility. Many previous studies have relied on non-registered or partially matched histopathology, limiting the precision of lesion-level validation, and interobserver variability has seldom been systematically evaluated.

In this study, we aim to evaluate the diagnostic performance of PSMA-PET/mpMRI for local staging of de novo prostate cancer and to compare it directly with standalone mpMRI and PSMA-PET obtained from the same hybrid examination. By using accurately registered whole-mount histopathology as the reference standard, we enable detailed lesion-by-lesion assessment of detection and staging accuracy. In addition, by assessing interobserver variability, we provide an estimate of the consistency and reliability of clinical staging, including the evaluation of T-stage, extraprostatic extension, and seminal vesicle invasion.

## Materials and methods

### Study participants

**Fig. 1:**
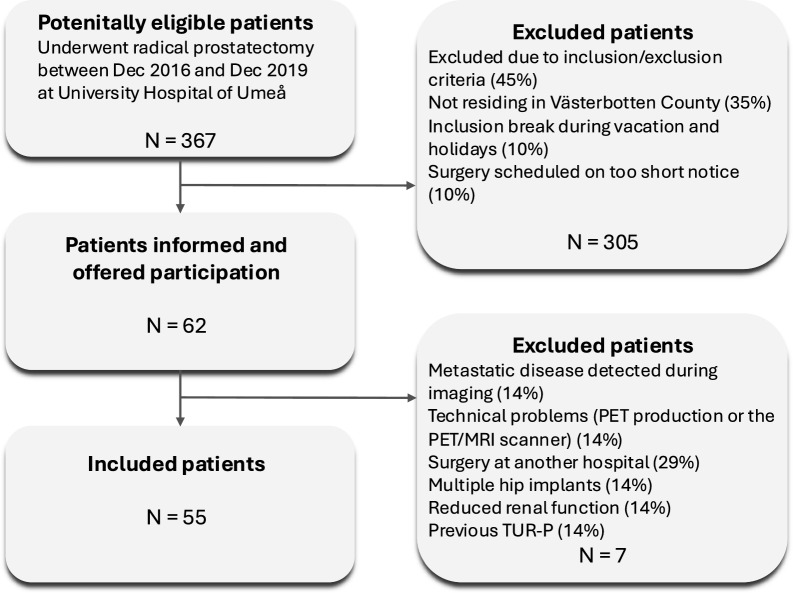
STARD diagram of patient inclusion

Between December 2016 and December 2019, 55 consecutive patients with intermediate- to high-risk prostate cancer scheduled for robot-assisted laparoscopic radical prostatectomy at University Hospital of Umeå were prospectively enrolled, as detailed in the STARD diagram (Fig. 1). Ethical approval was obtained from the Regional Ethics Board (DNR: 2016-220-31M) and the Swedish Medical Products Agency (EudraCT number: 2015–005046-55). Prior to surgery, all participants underwent a [^68^Ga]PSMA-PET/mpMRI to correlate in-vivo imaging findings with histological outcomes. This data has been used in previously published analysis [11, 23–26].

Inclusion criteria included ISUP grade ≥2, at least 2 months since last biopsy of the prostate, age >18 years and written informed consent. Exclusion criteria comprised contraindications to MRI or PET, World Health Organization performance status >1, impaired renal function, treatment with neoadjuvant/concomitant anti-testosterone treatment, TUR-P within 6 months, creatinine clearance <30 ml/min, metastatic prostate cancer and other known malignancy (except for basal cell carcinoma of the skin or malignancies with a progression-free survival of >10 years).

### Data acquisition

PSMA-PET imaging was performed simultaneously with mpMRI using a GE Signa 3 T PET/MRI scanner (GE Healthcare, Waukesha, WI, USA) with a total acquisition time of 41 minutes. PET-data was reconstructed with the high resolution iterative SharpIR algorithm (GE Healthcare, Waukesha, WI, USA). Patients received an intravenous injection of [^68^Ga]PSMA-11 at a dose of 2 MBq/kg, administered 60 minutes prior to imaging.

The mpMRI protocol included T2-weighted imaging in axial, coronal, and sagittal planes, as well as axial T1-weighted imaging. Diffusion-weighted imaging (DWI) was performed with b-values 0, 200, and 1000 s/mm^2^. Dynamic contrast-enhanced (DCE) imaging was acquired using 15 ml of Gd-DOTA (DOTAREM, Guerbet, Villepinte, France). The DCE acquisition was conducted over 8 minutes using a fast spoiled gradient-echo sequence with a frame acquisition time of 9.6 seconds. An apparent diffusion coefficient (ADC) map was generated by the MRI scanner software based on the DWI data. Details of PET and MRI sequence characteristics have been published previously [18].

For anatomical background during PSMA-PET evaluation, a synthetic CT was derived from a CT scan and deformably registered to the transverse large field-of-view T2-weighted MRI sequence.

### Imaging review

Two radiologists independently evaluated the mpMRI, while two nuclear medicine physicians assessed the PSMA-PET as presented earlier by Sandgren et al. [24]. For the hybrid PSMA-PET/mpMRI review, a new set of readers in two separate teams, each comprising one radiologist and one nuclear medicine physician, performed a consensus evaluation. The qualified reviewers (>5 years of experience) were blinded to clinical data, histopathology, and patient outcomes, with the only available information being the study’s inclusion criteria. The MRI reviewers independently assessed the mpMRI using the web-based DICOM viewer and reporting system from Collective Minds AB (www.cmrad.com). Meanwhile, the nuclear medicine physicians independently evaluated the PSMA-PET on the GE Advanced Workstation Server (AWS, GE Healthcare, Waukesha, WI, USA). The hybrid teams conducted their assessments of PSMA-PET/mpMRI in Hermes (Hermes Medical Solutions, Sweden) and Sectra PACS (Sectra AB, Sweden).

The evaluations were performed lesion-wise and included: location of the lesion (according to PI-RADS v2.1 sector map), classification of T-stage (according to TNM), and likelihood of EPE and SVI (Likert scale).

### Reference standard

After radical prostatectomy, the prostatic specimen was labelled for anatomical orientation and placed into a custom-made 3D-printed mold to prevent post-excision deformation. Before formalin fixation, the specimen was scanned ex-vivo using a T2-weighted MRI sequence. The specimen was then sectioned into 5 mm slices, paraffin-embedded, and microtomed into 5 µm slices, which were subsequently stained with H&E for histological examination. Tumourous regions were delineated using NDP.view2 (Hamamatsu Photonic K.K., Japan) under the supervision of a qualified pathologist with >30 years of experience, who also graded the lesions based on the ISUP system [27]. Tumour delineations that overlapped in the z-direction, in consecutive slices, were considered as the same lesion.

The histological slices were aligned to the ex-vivo T2-weighted MRI images using a 2D affine registration method. A rigid and deformable 3D registration was thereafter applied to align the histology with the in-vivo imaging. The software MICE Toolkit (Hero Imaging AB, Sweden) was used for the registrations and the method resulted in a median in-plane error of 1.7 mm. A detailed description of the registration workflow has been presented previously by Sandgren et al. [23].

In addition, pathological anatomical diagnosis (PAD) was collected for each patient. This report included the pathological stage for each patient: ISUP-grade, the pathological T-stage (pT-stage), SVI (yes/no), and EPE (yes/no).

### Lesions based analysis (detection analysis)

Tumour localization was mapped using the PI-RADS v2.1 sector map, specifying region, side, and zone [10]. A lesion was classified as a true positive (TP) when its reported location matched the histology-confirmed tumour site. Minor discrepancies in reported locations were resolved through visual inspection and adjusted if considered a match. False positives (FP) were lesions identified by reviewers without histological confirmation, while false negatives (FN) were lesions detected in histology but missed by reviewers. True negative (TN) cases were not included in the analysis.

### Patient based analysis (clinical staging analysis)

For patient-based analyses, the highest (worst) value from the lesion-based analysis was used, reflecting what would be reported in a clinical radiological assessment. These findings were then compared to the reference standard PAD. Tumours were staged according to the radiological TNM staging convention [3]. The likelihood of SVI and EPE was assessed using a 5-point Likert scale ranging from highly unlikely, unlikely, equivocal, likely to highly likely.

### Statistical analysis

Mean values between observers of the same modality were reported for detection rate, T-staging, EPE, and SVI, along with union results (correctly identified lesion or accurate classification by at least one observer). False discovery rates (FDR) were calculated as the number of FP divided by the total of reported lesions (TP + FP). Sensitivity and specificity values for EPE and SVI were calculated on dichotomized data where Likert scores of 1–3 were classified as negative and scores of 4–5 were considered positive. Differences in union detection rates and clinical staging between modalities were assessed using a two-sided McNemar test, with statistical significance set at p < 0.05. Interobserver reliability for detection rates and T-stage was evaluated using the index of specific agreement, as described by Shih et al. [28], while Cohen’s kappa statistics was used to assess interobserver reliability for EPE and SVI [29].

## Results

**Fig. 2:**
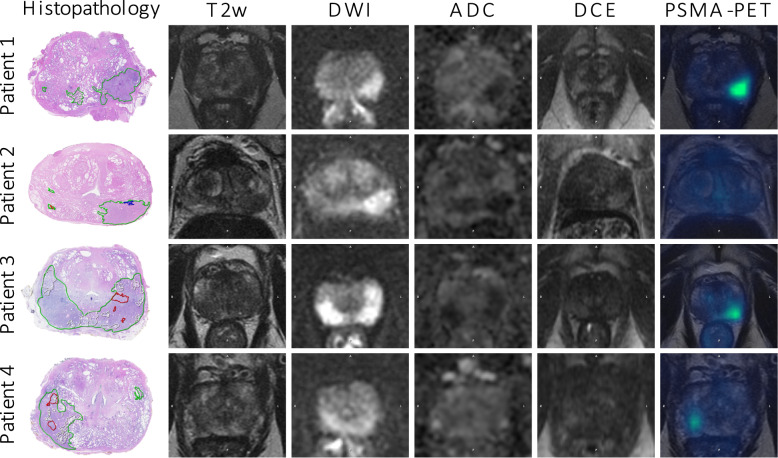
Images from four patients examined with [^68^Ga]PSMA-PET/mpMRI with corresponding histopathology slices (tumour lesions are delineated in green, Gleason grade 4 areas in red, Gleason grade 3 areas in blue and normal tissue in white). The mpMRI images include T2w sequence, DWI (b = 1000s/mm^2^), ADC map and early DCE. PSMA-PET with intensity range SUV = 10. In patient 1, all readers detected the dominant lesion (Gleason score 4+4). In patient 2, both MRI and hybrid readers detected the dominant lesion (Gleason score 4+3), but it was missed by the PSMA-PET readers. In patient 3, all readers detected the lesion (Gleason score 3+4). In patient 4, the dominant lesion (Gleason score 3+4) was detected by both PSMA-PET and hybrid readers, but it was missed by the MRI readers

After radical prostatectomy of the 55 included patients, 130 tumour lesions were observed by the pathologist at the histopathology evaluation. Lesion volumes ranged between 0.05–10.8 ml, with a median volume of 0.2 ml and a mean volume of 0.7 ml. Patient characteristics are presented in Table 1. Fig. 2 presents examples from four study participants, illustrating the in-vivo images with corresponding histopathology slice.

**Table 1:** Characteristics of the 55 included study participants

Characteristics	Median (min, max)
Age [years]	63 (45, 76)
PSA [ng/ml]	6.3 (2.9, 13.3)
Days between imaging and surgery	26 (2, 138)
Injected activity PSMA [MBq]	163 (121, 201)
**Post RP ISUP**	**N (%)**
2	29 (52.7)
3	17 (30.9)
4	5 (9.1)
5	4 (7.3)
**pT status**	
T2	25 (45.5)
T3	30 (54.5)
**pN status**	
Not removed	44 (80.0)
Lymph nodes removed without metastasis	9 (16.4)
Lymph nodes removed with metastasis	2 (3.6)
**Surgical margin**	
Positive	14 (25.5)
Negative	41 (74.5)
**Seminal vesicle involvement**	
None	51 (92.7)
Right	0 (0)
Left	2 (3.6)
Both	2 (3.6)

N=number of patients, RP=radical prostatectomy.

### Detection rate

**Fig. 3:**
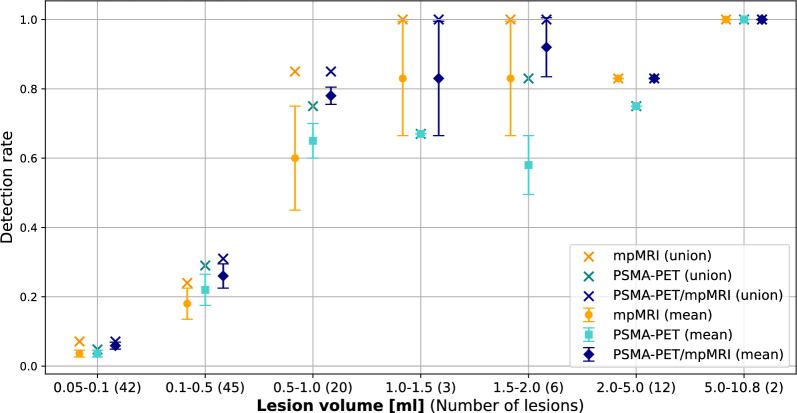
Detection rate stratified by lesion volume. Results are shown as the mean between the two observers, with error bars representing individual observer detection rate. Union detection rates, defined as lesions identified by at least one observer, are also presented

**Fig. 4:**
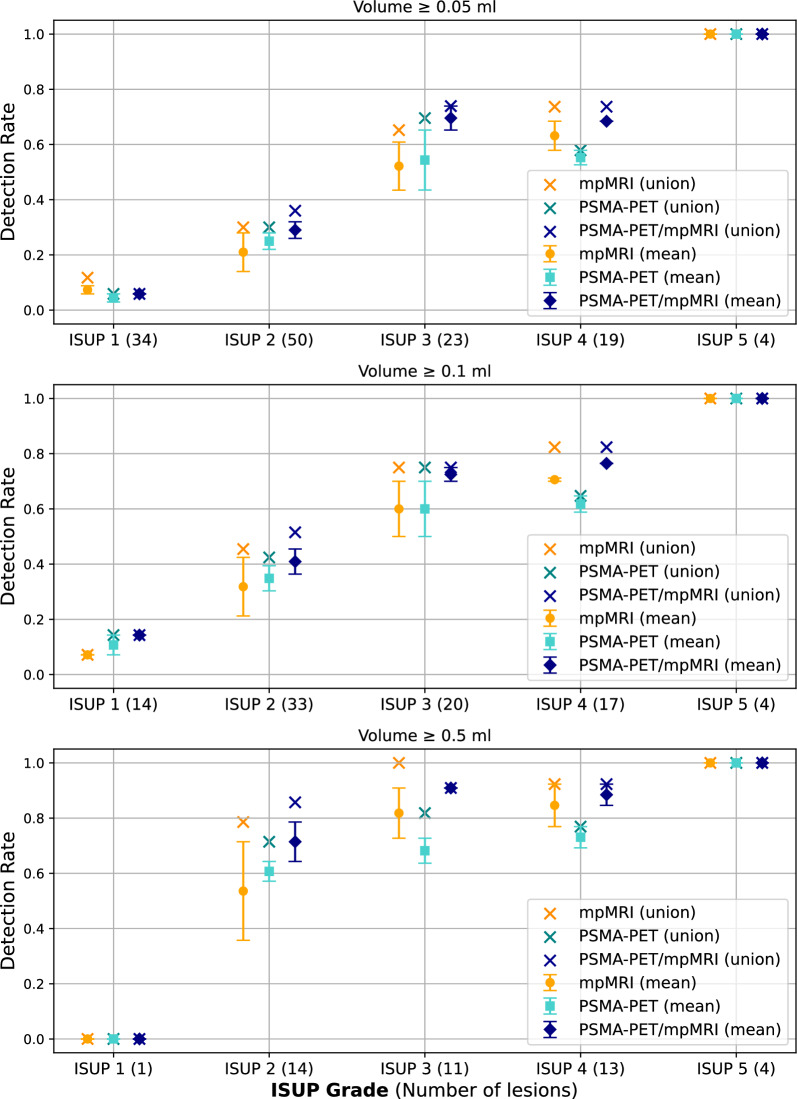
Detection rates stratified by ISUP grade and three volume thresholds (≥0.05 ml, ≥0.1 ml, and ≥0.5 ml). Results are shown as the mean between the two observers, with error bars representing individual observer detection rate. Union detection rates, defined as lesions identified by at least one observer, are also presented

Detection rates categorized into various volume thresholds are shown in Fig. 3, while Fig. 4 illustrates detection rates based on ISUP grade across three volume thresholds: ≥0.05 ml, ≥0.1 ml, and ≥0.5 ml.

For csPC lesions (≥0.5 ml, ≥ISUP 2; 42 in total), mean detection rates were highest with PSMA-PET/mpMRI at 85% (35.5/42), followed by mpMRI at 75% (31.5/42) and PSMA-PET at 70% (29.5/42). Union detection rates, which represent lesions identified by at least one observer, were 90% (38/42) for both PSMA-PET/mpMRI and mpMRI, while PSMA-PET had a lower rate of 79% (33/42).

For lesions ≥ISUP 2 with a volume between 0.1 ml and 0.5 ml (32 in total), mean detection rates decreased but remained highest for PSMA-PET/mpMRI at 30% (9.5/32), compared to 22% (7/32) for mpMRI and 27% (8.5/32) for PSMA-PET. Union detection rates were 38% (12/32) for PSMA-PET/mpMRI, 31% (10/32) for mpMRI and 34% (11/32) for PSMA-PET.

For all histopathological confirmed lesions (≥0.05 ml, 130 in total), mean detection rates were highest for PSMA-PET/mpMRI at 38% (49.5/130), followed by mpMRI and PSMA-PET, both at 32% (41/130). Union detection rates were 42% (55/130) for PSMA-PET/mpMRI, 40% (52/130) for mpMRI, and 37% (48/130) for PSMA-PET. The lowest FDR was found for PSMA-PET/mpMRI at 8% (range 6–10%), followed by mpMRI at 15% (range 11–20%) and PSMA-PET at 12% (range 10–14%).

Of the 130 histopathological confirmed lesions (≥0.05 ml), 110 were at least partially located in the peripheral zone. Among these, 84 lesions were classified as ≥ISUP 2, with mean detection rates of 52% (44/84) for PSMA-PET/mpMRI, 44% (37/84) for mpMRI, and 43% (36.5/84) for PSMA-PET. Union detection rates for these lesions were 57% (48/84), 54% (45/84), and 50% (42/84), respectively. For the 12 lesions classified as ≥ISUP 2 and located outside the peripheral zone, mean detection rates were 29% (3.5/12) for PSMA-PET/mpMRI, 12% (1.5/12) for mpMRI, and 25% (3/12) for PSMA-PET, while union detection rates were 42% (5/12), 25% (3/12), and 33% (4/12), respectively.

### T-staging

During the evaluation, reviewers determined the T-stage for all detected lesions. The highest (worst) grade from the lesion-based analysis was chosen for each patient and compared to histopathology. Within the patient cohort, 25 patients had pT2 tumours, while 30 had pT3 tumours. The results of the radiological staging (mean and union), categorized by pT2 and pT3, are shown in Table 2.

**Table 2:** Results of the clinical T-staging of the study participants

		pT2	pT3	All stages
PSMA-PET/mpMRI	Mean	70% (17.5/25)	35% (10.5/30)	51% (28/55)
	Union	80% (20/25)	57% (17/30)	67% (37/55)
mpMRI	Mean	54% (13.5/25)	33% (10/30)	43% (23.5/55)
	Union	76% (19/25)	43% (13/30)	58% (32/55)
PSMA-PET	Mean	58% (14.5/25)	7% (2/30)	30% (16.5/55)
	Union	64% (16/25)	13% (4/30)	36% (20/55)

The table represents the fraction of correctly staged patients to the total number of classified stages (pT2, pT3 or both). Results are presented as the mean of the two observers and as union results, defined as cases correctly classified by at least one observer.

T-stage prediction accuracy varied depending on tumour stage. For pT2 tumours, mean accuracy was 70% with PSMA-PET/mpMRI, 54% with mpMRI and 58% with PSMA-PET. For pT3 tumours, accuracy decreased to 35% for PSMA-PET/mpMRI, 33% for mpMRI and 7% for PSMA-PET.

### EPE and SVI-prediction

**Figure 5:**
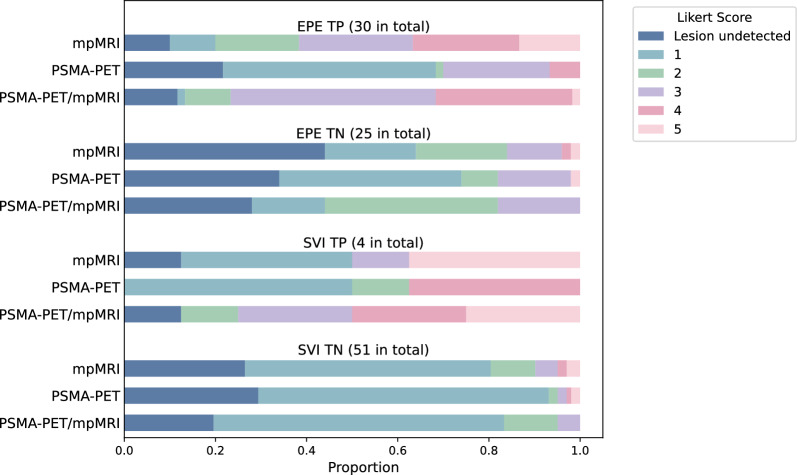
Proportion of assigned Likert score stratified by TP and TN cases for EPE and SVI

The reviewers reported the likelihood of EPE and SVI using a 5-point Likert scale, where scores of 1–3 were considered negative and 4–5 were considered positive. Within the patient cohort, 30 patients had EPE, and 4 had SVI. The proportion of assigned Likert scores for TP and TN cases are presented in Fig. 5.

Mean sensitivity for EPE, was 32% for PSMA-PET/mpMRI, 37 % for mpMRI and 7% for PSMA-PET, with a specificity of 100%, 96% and 98% respectively. In union analysis, the sensitivity was 57% for PSMA-PET/mpMRI, 50% for mpMRI and 13% for PSMA-PET, with a specificity of 100%, 96% and 96% respectively.

For SVI, mean sensitivity was 50% for PSMA-PET/mpMRI, while it was 38% for both mpMRI and PSMA-PET. Mean specificity was 100% for PSMA-PET/mpMRI, 95% for mpMRI and 97% for PSMA-PET. In union analysis, the sensitivity was 50% for PSMA-PET/mpMRI, 50% for mpMRI and 75% for PSMA-PET, with a specificity of 100%, 90% and 96% respectively.

### Modality based differences in diagnostic accuracy (McNemar test)

For lesions ≥0.1 ml, the McNemar test showed no statistically significant differences in detection rates between the imaging modalities. In terms of classification accuracy, the McNemar test revealed significantly higher accuracy for pT3 and EPE when comparing both PSMA-PET/mpMRI and mpMRI to PSMA-PET alone, with PSMA-PET performing inferior in both comparisons. P-values are presented in Table 3.

**Table 3:** P-values from McNemar tests comparing union detection rate (DR) and union classification accuracy for pT2, pT3, EPE, and SVI between modalities

	DR	pT2	pT3	EPE	SVI
mpMRI vs PSMA-PET	0.63	0.58	0.02*	0.006*	1.00
PSMA-PET/mpMRI vs mpMRI	0.51	1.00	0.29	0.68	1.00
PSMA-PET/mpMRI vs PSMA-PET	0.18	0.22	0.001*	0.001*	1.00

Union results represent lesions correctly identified or classifications accurately assigned by at least one observer. *p < 0.05 (statistically significant).

### Interobserver agreement

Interobserver agreement for detecting lesions ≥0.1ml, measured by the index of specific agreement, was 89% for PSMA-PET/mpMRI, 76% for mpMRI and 84% for PSMA-PET. For lesions ≥0.5 ml, agreement increased to 93% for PSMA-PET/mpMRI, 79% for mpMRI and 88% for PSMA-PET. For pT2 tumours, the index of specific agreement was 86% for PSMA-PET/mpMRI, 59% for mpMRI and 90% for PSMA-PET. For pT3 tumours, the corresponding values were 38%, 70%, and 0%, respectively.

Interobserver reliability for EPE, evaluated using Cohen’s kappa, indicated slight agreement for PSMA-PET/mpMRI (κ = 0.13), moderate agreement for mpMRI (κ = 0.55) and no agreement for PSMA-PET (κ = 0). For SVI, kappa values showed perfect agreement for PSMA-PET/mpMRI (κ = 1.00), substantial agreement for mpMRI (κ = 0.66) and no agreement for PSMA-PET (κ = 0).

## Discussion

In this study we compared the performance of PSMA-PET/mpMRI, mpMRI and PSMA-PET in detecting and staging intraprostatic lesions. The standalone modalities (mpMRI and PSMA-PET) were acquired from the hybrid examination which allows this study to directly compare the diagnostic performance of the three modalities. All modalities were reviewed independently by two (mpMRI and PSMA-PET) or four (PSMA-PET/mpMRI) qualified reviewers with experience in the field of prostate cancer imaging. Our results shows that PSMA-PET/mpMRI had higher mean detection rates than mpMRI alone, while PSMA-PET alone had the lowest performance. Although union detection rates for csPC lesions were equal between PSMA-PET/mpMRI and mpMRI, the hybrid approach still demonstrated an advantage by reducing the FDR, suggesting its potential to minimize the risk of misdirecting biopsies and improve diagnostic accuracy.

These findings are consistent with those reported by Eiber et al. [18], who demonstrated that PSMA-PET/mpMRI improves the accuracy for intraprostatic tumour localization compared to mpMRI and PSMA-PET alone, correctly identifying lesions in 98% of cases versus 66% for mpMRI and 92% for PSMA-PET. Similarly, Hicks et al. [17] reported that PSMA-PET/mpMRI achieved a higher sensitivity than mpMRI for the detection of intraprostatic tumours, also demonstrating the added value of hybrid imaging. Previous research also indicates that combining PSMA-PET with MRI may improve the accuracy of targeted biopsies [30].

In our study, the index of specific agreement for lesion detection was higher for PSMA-PET/mpMRI compared to mpMRI and PSMA-PET, indicating greater consistency between observers. Some bias may however be introduced by the fact that the PET/mpMRI teams and the PSMA-PET readers were from the same institutions, respectively. Potentially this can explain the slightly lower agreement seen within the mpMRI-team, with observers from different institutions.

For T-stage prediction, PSMA-PET/mpMRI demonstrated the highest mean accuracy, particularly for pT2 tumours, with an accuracy of 70%, compared to 54% for mpMRI and 58% for PSMA-PET, indicating that the combined modality offers improved diagnostic performance in local staging. Similarly, Grubmüller et al. also reported high accuracy rates for PSMA-PET/mpMRI (85% for T2, 79% for T3a, and 94% for T3b tumours) [22]. In the prediction of EPE, our mean sensitivity was generally low across all modalities, ranging from 7% to 37%, while the specificity remained high (96–100%). In detecting SVI, PSMA-PET/mpMRI showed higher mean sensitivity and specificity compared to mpMRI and PSMA-PET. However, with only four SVI cases in the cohort, drawing definitive conclusions are difficult.

A meta-analysis by Rooij et al. [31], including 75 studies, reported higher sensitivity for mpMRI, compared to our study, with a sensitivity of 57% and specificity of 91% for EPE, and 58% sensitivity and 96% specificity for SVI. Notably, this analysis includes various assessment criteria for EPE and SVI. When using a Likert score of ≥3 as the threshold for a positive finding on our data, sensitivity improved and became more comparable to the values reported by Rooij et al., with the cost of slightly lower specificity. Specifically, EPE mean sensitivity increased to 77% for PSMA-PET/mpMRI, 62% for mpMRI and 30% for PSMA-PET, with corresponding mean specificity of 82%, 84% and 82% respectively. For SVI, mean sensitivity improved to 75% for PSMA-PET/mpMRI, 50% for mpMRI and remained at 38% for PSMA-PET, with a mean specificity of 95%, 90% and 95% respectively.

A key strength of our study is the use of registered whole-mount histopathology as reference standard. This approach enabled evaluation of detection performance across different tumour volume thresholds, including small-volume disease, and allowed assessment of all tumour foci within the prostate rather than focusing solely on the index lesion.

Another important strength is the use of simultaneous PSMA-PET/mpMRI acquisition, which eliminates potential misregistration caused by patient repositioning or time delays between sequential scans, thereby improving anatomical and functional correspondence. While our study utilized a hybrid PET/MRI system, the more common clinical scenario involves separate PET and MRI acquisitions, followed by co-registration. When combining evaluations from one PET and one MRI observer, the mean detection rate for csPC lesions (≥0.5 ml, ISUP ≥2) was 89% (range: 79–93%), compared to 85% (range: 83–86%) for the hybrid approach.

One challenge in performing a patient-based analysis using lesion-based data is the potential mismatch between radiological and histopathological findings, particularly in multifocal disease. When multiple lesions are present, there is a risk that the radiological assessment may correspond to a different lesion than the one identified in the histopathological report. To minimize this discrepancy, the lesion with the highest risk characteristics was selected from the radiological report in cases where multiple lesions were detected. This approach aimed to better simulate a clinically relevant patient-based radiological assessment.

There are additional challenges in performing an in-depth radiological evaluation, including comparing graded judgements (Likert scale) to binary information provided by the PAD. For instance, in PAD extraprostatic extensions was defined as true independent of the extent. Another challenge is to provide the reviewer with equal information before the review process. To enhance consistency in the evaluations, all reviewers were briefed individually (or in pairs), and we used a structured digital questionnaire to collect the data with predefined units and relevant information to minimize the risk of type errors and misunderstandings. Despite these measures, variability in reader performance was observed, potentially due to the tendency toward downgrading lesions when in doubt and the inherent subjectivity of Likert scale assessments.

A limitation of this study is that the MRI scanner used, which was state-of-the-art when the study began in 2016, has since been surpassed by newer systems offering substantially higher image quality. The scanning protocol used were implemented before the release of the PI-RADS v2.1 guideline which specifies a DWI slice thickness of 3 mm. For this study a slice thickness of 5 mm was used for the DWI to facilitate easy correlation with the histopathology sectioning, potentially reducing the sensitivity for small lesion detection.

This study focused on performance of PSMA-PET/mpMRI in local staging of prostate cancer with histopathological verification. From a clinical point of view, metastasis assessment with a whole-body protocol is required for comprehensive staging evaluation. This is currently done with CT and PET/CT but may also be an option in PET/MRI with extended protocol, which can be considered for future PET/MRI studies for planned clinical implementation.

## Conclusion

This study indicates that PSMA-PET/mpMRI provides added value in the assessment of localized prostate cancer compared to standalone mpMRI and PSMA-PET. It demonstrated higher and more consistent performance in lesion detection and staging without increasing false-positive findings, thereby reducing the risk of misdirected biopsies and suboptimal treatment decisions.

## Data Availability

The dataset generated and analysed during the current study are not publicly available as this was not specified in the informed consent and ethical approval but are in selected parts available from the corresponding author on reasonable request.

## Abbreviation

ADC: Apparent diffusion coefficient
csPC: Clinically significant prostate cancer
CT: Computed tomography
DCE: Dynamic contrast-enhanced
DWI: Diffusion-weighted imaging
EPE: Extraprostatic extension
FDR: False discovery rate
FN: False negative
FP: False positive
mpMRI: Multiparametric magnetic resonance imaging
PAD: Pathological anatomical diagnosis
PI-RADS: Prostate imaging reporting and data system
PET: Positron emission tomography
PSMA: Prostate-specific membrane antigen
SVI: Seminal vesicle invasion
TN: True negative
TP: True positive
